# Biochemical alterations in inflammation, oxidative stress, and urinary biomarkers associated with renal damage in systemic lupus erythematosus

**DOI:** 10.5937/jomb0-62936

**Published:** 2026-05-22

**Authors:** Zhang Rui, Yiyang Yu, Guangxu Liu, Zhongqiu Luan

**Affiliations:** 1 The First Affiliated Hospital of Heilongjiang University of Traditional Chinese Medicine, Department of Nephropathy, Harbin, China

**Keywords:** systemic lupus erythematosus, renal damage, biochemical markers, oxidative stress, inflammatory cytokines, urinary biomarkers, sistemski eritematozni lupus, oštećenje bubrega, biohemijski markeri, oksidativni stres, inflamatorni citokini, urinarni biomarkeri

## Abstract

**Background:**

Renal damage is a major complication of systemic lupus erythematosus (SLE) and is closely linked to biochemical disturbances involving inflammation, oxidative stress, and renal tubular injury. However, the biochemical signatures that differentiate SLE patients with and without renal involvement remain insufficiently characterized. To evaluate the biochemical profiles of inflammatory cytokines, oxidative stress markers, and urinary renal-injury biomarkers in SLE patients with and without renal damage, and to explore their associations with microbial alterations.

**Methods:**

Sixty-four SLE patients were classified into a renal damage group (n = 36; positive urine protein) and an SLE-only group (n = 28; negative urine protein). Serum cytokines (TGF-R, IL-4, IL-17, IL-1P) and oxidative stress indicators (SOD, T-AOC, MDA) were quantified using ELISA. Urinary microprotein, microglobulin, and N-acetyl-P-D-glucosaminidase (NAG) were used as biochemical markers of renal injury. Oral and intestinal microbial profiles were analyzed by sequencing, and correlations between biochemical markers and microbial alterations were assessed.

**Results:**

SLE patients with renal damage showed significantly elevated urinary microprotein, microglobulin, and NAG (all p &lt; 0.001). Inflammatory cytokines were markedly increased in the renal damage group (TGF-P, IL-4, IL-17, IL-1P; all p &lt; 0.05), whereas oxidative stress capacity was significantly reduced (SOD, T-AOC, MDA; all p &lt; 0.05). Several microbial taxa correlated positively or negatively with key biochemical markers, suggesting potential metabolic-immune interactions contributing to renal injury.

**Conclusions:**

Renal damage in SLE is characterized by distinct biochemical abnormalities involving intensified inflammation, impaired antioxidant defenses, and elevated urinary renal-injury biomarkers. These biochemical changes, together with specific microbial shifts, may contribute to the progression of SLE-related renal impairment and hold diagnostic value for early biochemical screening.

## Abstract (sr)

**Uvod:**

Ostećenje bubrega je glavna komplikacija sistemskog eritematoznog lupusa (SLE) i usko je povezano sa biohemijskim poremećajima koji uključuju upalu, oksidativni stres i oštećenje bubrežnih tubula. Međutim, biohemijski potpisi koji razlikuju pacijente sa SLE sa i bez oštećenja bubrega ostaju nedovoljno okarakterisani. Cilj je procena biohemijskih profila inflamatornih citokina, markera oksidativnog stresa i biomarkera oštećenja bubrega u urinu kod pacijenata sa SLE sa i bez oštećenja bubrega, i istraživanje njihove povezanosti sa mikrobnim promenama.

**Metode:**

Šezdeset četiri pacijenta sa sistemskim lupusom eritematozusom (SLE) klasifikovano je u grupu sa oštećenjem bubrega (n = 36; pozitivan protein u urinu) i grupu samo sa SLE (n = 28; negativan protein u urinu). Serumski citokini (TGF-P, IL-4, IL-17, IL-1p) i indikatori oksidativnog stresa (SOD, T-AOC, MDA) kvantifikovani su pomoću ELISA testa. Urinarni mikroprotein, mikroglobulin i N-acetil-P-D-glukozaminidaza (NAG) korišćeni su kao biohemijski markeri oštećenja bubrega. Oralni i crevni mikrobni profili analizirani su sekvenciranjem, a procenjene su i korelacije između biohemijskih marker.

## Introduction

Systemic lupus erythematosus (SLE) is a chronic autoimmune disorder characterized by persistent immune activation, autoantibody production, and multisystem involvement [1]. Among its various clinical manifestations, renal damage (lupus nephritis) is one of the most severe complications and a major determinant of long-term prognosis. The onset and progression of SLE-associated renal injury are closely related to complex biochemical disturbances, including dysregulated inflammatory signaling, oxidative stress imbalance, and biochemical indicators of renal tubular dysfunction [2].

Increasing evidence suggests that inflammatory cytokines - including transforming growth factor-β (TGF-β), interleukin-4 (IL-4), IL-17, and IL-1β - participate directly in glomerular injury, mesangial proliferation, and tubular interstitial inflammation [3]. At the same time, oxidative stress pathways play a pivotal role in accelerating tissue injury, with reduced antioxidant capacity (e.g., decreased superoxide dismutase [SOD] and total antioxidant capacity [T-AOC]) enhancing lipid peroxidation and renal cellular injury, as reflected by increased levels of malondialdehyde (MDA) [4][5]. From a biochemical standpoint, these abnormalities represent critical molecular events underlying SLE progression [6].

Urinary biochemical indicators, such as microprotein, microglobulin, and N-acetyl-β-D-glucosaminidase (NAG), are sensitive markers of early renal damage and tubular dysfunction [7]. Their evaluation provides clinically valuable information for biochemical screening and monitoring of renal involvement in SLE. However, the integrated biochemical profile that distinguishes SLE patients with renal impairment from those without remains insufficiently defined [8][9].

Recent studies also highlight interactions between microbial dysbiosis and host biochemical processes. Altered oral and intestinal flora may modulate inflammatory cytokines, oxidative stress signaling, and metabolic pathways, thereby influencing SLE disease activity [10][11]. Nevertheless, the biochemical consequences of microbiota changes in SLE-related renal injury require further clarification.

The present study aimed to investigate the biochemical signatures of inflammation, oxidative stress, and renal-injury biomarkers in SLE patients with renal damage. By combining laboratory biochemical analyses with microbial profiling, we sought to delineate the biochemical changes accompanying renal involvement and provide evidence for their potential diagnostic value in clinical biochemistry practice.

## Materials and methods

### Patients enrolled in this study

A total of 64 SLE patients recently treated in our hospital were taken as research objects, and divided into renal damage group (n = 36, patients with positive urine protein) and SLE group (n=28, patients with negative urine protein). All cases were in compliance with the 1982 revised criteria for the classification of systemic lupus erythematosus by American College of Rheumatology. In SLE group, there were 2 males and 26 females, with an average age of (34.12±3.84) years old, while in renal damage group, there were 3 males and 33 females, with an average age of (33.65±4.65) years old. There were no statistically significant differences in general data such as gender and age between the two groups (*p*>0.05). This study was approved by the ethics committee of The First Affiliated Hospital of Heilongjiang University of Traditional Chinese Medicine. Signed written informed consents were obtained from all participants before the study.

### Collection of fasting venous blood samples

Fasting venous blood samples were collected in the early morning. After centrifugation at 3,000 rpm for 10 minutes, serum was separated, aliquoted, and immediately stored at -80°C until analysis, ensuring no more than one freeze-thaw cycle. Twenty-four-hour urine samples were collected under standardized pre-analytical conditions. Specifically, all urine was kept at 4°C throughout the collection period, transported to the laboratory within 1 hour, centrifuged at 3,000 rpm for 10 minutes, and the resulting supernatant was aliquoted and stored at -80°C until biochemical testing. No urine specimen underwent more than one freeze-thaw cycle. All biochemical measurements were performed in the Clinical Biochemistry Laboratory of our institution under routine internal quality-control procedures.

### Inflammatory cytokines

Serum inflammatory cytokines, including transforming growth factor-β (TGF-β), interleukin-4 (IL-4), interleukin-17 (IL-17), and interleukin-1β (IL-1β), were quantified using commercially available enzyme-linked immunosorbent assay (ELISA) kits (Manufacturer: Beyotime, Country: China) following standardized biochemical procedures. Each assay included a full calibration curve generated from serially diluted standards, with the coefficient of determination (R^2^) consistently above 0.98. The analytical sensitivity levels were within the ranges provided by the manufacturer, and both intra-assay and interassay coefficients of variation remained below 10-12%. All samples were measured in duplicate, and mean values were used for final analyses. Strict attention was paid to pre-analytical factors, including serum separation time, storage temperature, and avoidance of hemolysis, to ensure biochemical reliability.

### Oxidative stress markers and urinary biochemical indicators

Biochemical indicators of oxidative stress, including superoxide dismutase (SOD), total antioxidant capacity (T-AOC), and malondialdehyde (MDA), were determined using standardized colorimetric assay kits (Manufacturer: Beyotime, Shanghai, China). Assays were performed in accordance with reagent specifications, and internal quality-control samples were included in each batch to verify assay precision. Urinary biochemical indicators reflecting renal tubular injury - urinary microprotein, microglobulin, and N-acetyl-β-D-glucosaminidase (NAG) - were measured using automated biochemical analyzers (Model and Manufacturer: [insert]). These biomarkers were selected due to their high sensitivity for early renal damage and established clinical utility in renal biochemical assessment.

### Oral and intestinal microflora analysis

Oral microflora samples were collected using sterile swabs after at least four hours of fasting and thorough mouth rinsing to remove food residue. Fresh mid-segment stool samples (approximately 5 g) were collected for intestinal flora analysis. Microbial DNA was extracted using standard purification kits, and the V3-V4 region of the bacterial 16S rRNA gene was amplified by polymerase chain reaction. Sequencing was carried out using the Illumina MiSeq platform, and subsequent bioinformatic analyses included quality filtering, operational taxonomic unit clustering, and taxonomic assignment using established reference databases. Although microbial profiling was included, its interpretation was focused primarily on biochemical relevance, particularly the potential influence of microflora on inflammatory and oxidative biochemical pathways.

### Statistical analysis

All statistical analyses were performed using SPSS 23.0. Continuous variables were expressed as mean ± standard deviation. Before applying parametric tests, data distribution was assessed using the Shapiro-Wilk test, and homogeneity of variance was evaluated using Levene's test. Variables meeting normality and variance assumptions were analyzed using independent-sample t-tests; otherwise, appropriate non-parametric methods were considered. Correlations between biochemical markers and microbial abundance were evaluated using Pearson correlation coefficients. A p-value < 0.05 was considered statistically significant.

## Results

### Biochemical indicators of renal injury

Significant biochemical differences in renal function were observed between the two groups. As shown in Table 1, patients in the renal damage group exhibited markedly elevated urinary microprotein, urinary microglobulin, and urinary N-acetyl-β-D-glucosaminidase (NAG) levels compared with the SLE group (all p < 0.001). These findings indicate substantial renal tubular dysfunction and provide biochemical evidence of renal injury associated with SLE.

**Table 1:** Differences in indexes associated with renal function between SLE group and renal damage group.

Group	n	Urinary microprotein <br>(mg/L)	Urinary microglobulin <br>(mg/L)	Urinary NAG <br>(U/L)
SLE group	28	26.74±3.85	43.85±4.51	23.15±2.48
Renal damage group	36	236.53±12.94	179.32±25.62	78.53±6.58
t		35.32	46.64	18.91
p		0.000	0.000	0.000

### Inflammatory cytokine profiles

**Figure 1:**
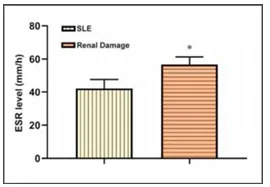
Changes in ESR (mm/h) between SLE group and renal damage group (*p < 0.05 vs. SLE group).

Inflammatory activity was clearly higher in patients with renal involvement. According to Table 2, serum concentrations of TGF-β, IL-4, IL-17, and IL-1β were all significantly increased in the renal damage group (p < 0.05 for each cytokine). Additionally, erythrocyte sedimentation rate (ESR) was elevated in these patients, as shown in Figure 1, supporting a state of intensified systemic inflammation.

**Table 2:** Differences in inflammatory factors between the two groups (mean ± SD; ng/L).

Group	n	TGF-β	IL-4	IL-17	IL-1β
SLE group	28	24.51±2.64	12.31±1.45	18.45±1.74	14.56±1.43
Renal damage group	36	33.43±3.29	18.75±2.45	22.45±2.94	16.53±2.13
*t*		8.65	7.59	9.34	5.84
*p*		0.032	0.038	0.021	0.047

### Oxidative stress biomarker alterations

Differences in oxidative stress levels were also evident. As presented in Table 3, the renal damage group demonstrated significantly reduced SOD and T-AOC levels, accompanied by lower MDA concentrations compared with the SLE group (all p < 0.05). This biochemical pattern reflects impaired antioxidant defenses and disruption of redox homeostasis in SLE patients with renal injury.

**Table 3:** Changes in oxidative stress level between SLE group and renal damage group (mean ± SD). Changes in ESR (mm/h) between SLE group and renal damage group (*p < 0.05 vs. SLE group).

Group	n	SOD (U/mL)	T-AOC (U/mL)	MDA (nmol/mL)
SLE group	28	98.23±6.48	9.43±2.74	8.22±0.85
Renal damage group	36	56.54±7.95	8.37±1.25	5.86±1.25
*t*		9.23	5.23	7.56
*p*		0.022	0.048	0.037

### Oral microflora composition

**Figure 2:**
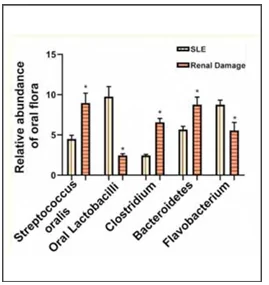
Relative abundance (%) of major oral microbial genera in SLE and renal-damage groups. (*p < 0.05 vs. SLE group).

Distinct shifts in oral flora were observed between the two groups. As illustrated in Figure 2, the renal damage group showed higher relative abundances of *Streptococcus oralis, Clostridium*, and *Bacteroidetes*, whereas *Lactobacilli* and *Flavobacterium* were significantly reduced (p < 0.05). These microbial alterations may contribute to biochemical changes in immune regulation.

### Intestinal microflora differences

**Figure 3:**
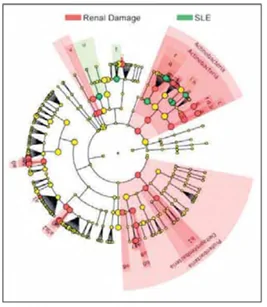
Analysis of intestinal flora in both groups.

**Figure 4:**
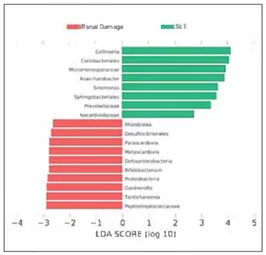
LDA score of intestinal flora in SLE group and renal damage group.

Analysis of intestinal flora revealed additional group-specific variations. According to Figure 3 and Figure 4, *Collinsella, Coriobacteriales, Micromonosporaceae, Asaccharobacter*, and *Sinomonas* were more abundant in the SLE group, while *Rhizobiales, Desulfovibrionales, Parascardovia, Metascardovia, Deltaproteobacteria*, and *Bifidobacterium* were enriched in the renal damage group. These findings suggest potential microbial influences on biochemical pathways related to inflammation and renal function.

### Correlations among intestinal flora

**Figure 5:**
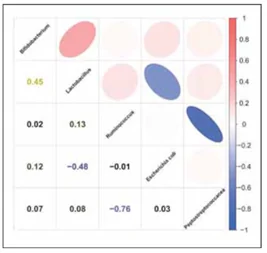
Pearson correlation analysis of intestinal flora.

Correlation analysis demonstrated significant associations between key microbial taxa. As shown in Figure 5, *Bifidobacterium* was positively correlated with *Lactobacillus* (r = 0.45, p = 0.001), while negative correlations were found between *Ruminococcus* and *Peptostreptococcacea* (r = -0.76, p = 0.000), and between *Escherichia coli* and *Lactobacillus* (r = -0.48, p = 0.021). These relationships highlight potential microbe-biochemical interactions that may modulate inflammatory and oxidative processes.

## Discussion

Renal damage is a severe and common complication of systemic lupus erythematosus (SLE), and its development is tightly linked to a complex network of biochemical abnormalities [12]. The present study demonstrated that SLE patients with renal involvement exhibit significant alterations in inflammation, oxidative stress, and urinary renal-injury biomarkers, together with characteristic changes in oral and intestinal microflora. These combined biochemical and microbial findings provide insight into the pathophysiological processes underlying SLE-related renal impairment.

The marked elevations in urinary microprotein, microglobulin, and N-acetyl-β-D-glucosaminidase (NAG) in the renal damage group reflect significant tubular injury and dysfunction [13]. Microprotein and microglobulin are highly sensitive biochemical markers of glomerular and tubular impairment, while NAG is a lysosomal enzyme released during early proximal tubular injury [14][15]. Their pronounced increases confirm substantial biochemical evidence of renal tissue damage and support their potential utility as early biochemical indicators in SLE patients at risk of renal involvement [16].

Inflammatory cytokines play critical roles in SLE pathogenesis, and our results show that TGF-β, IL-4, IL-17, and IL-1β levels were significantly elevated in patients with renal damage. These cytokines participate in key inflammatory and immunoregulatory pathways: IL-17 promotes neutrophil recruitment and sustained tissue inflammation; TGF-β contributes to renal fibrosis; IL-4 modulates T-helper cell differentiation; and IL-1β is a central mediator in inflammasome activation. Together with an elevated ESR, these findings illustrate that SLE-associated renal injury is accompanied by intensified biochemical inflammatory activity. This biochemical cytokine profile may also contribute to immune complex deposition and complement activation, accelerating renal structural damage.

Oxidative stress is another fundamental biochemical mechanism contributing to lupus nephritis. Compared with SLE patients without renal involvement, the renal damage group displayed significantly lower levels of SOD, T-AOC, and MDA. SOD and T-AOC are central antioxidant defense indicators, and their reduction reflects impaired capacity to neutralize reactive oxygen species. Although MDA is a lipid peroxidation product typically elevated during oxidative stress, its decrease may reflect biochemical imbalance or consumption during severe oxidative processes. Collectively, these changes suggest that compromised antioxidant capacity and disturbed redox homeostasis may drive renal cellular injury in SLE, possibly through mitochondrial dysfunction or enhanced lipid peroxidation [17].

In addition to biochemical abnormalities, characteristic alterations in oral and intestinal flora were identified. Changes in *Streptococcus oralis, Clostridium, Bacteroidetes*, and reduced *Lactobacilli* and *Flavobacterium* may influence oral immune activation and systemic cytokine levels [18]. More pronounced differences were observed in intestinal microflora, with specific genera enriched in the SLE group and others dominant in the renal damage group. Microbial metabolites such as short-chain fatty acids, lipopolysaccharides, and trimethylamine N-oxide (TMAO) are known to modulate oxidative stress, immune responses, and renal inflammation [19][20]. Although this study did not directly quantify microbial metabolites, the observed correlations between bacterial taxa - such as the positive association between *Bifidobacterium* and *Lactobacillus*, and negative associations involving *Ruminococcus* and *Escherichia coli* - suggest potential biochemical-microbial interactions contributing to disease progression.

Overall, these findings provide a comprehensive biochemical profile of SLE patients with renal involvement, highlighting the interplay among inflammatory cytokines, oxidative stress, renal-injury biomarkers, and microbiota alterations. The biochemical markers examined in this study may serve as useful laboratory indicators for early detection and monitoring of SLE-associated renal damage. Moreover, the microbial findings raise the possibility that microbiota-modulated biochemical pathways may influence renal pathology, which represents a potential area for future mechanistic and translational research.

Although microbial profiling was descriptive, several genera identified in this study have known biochemical influences on inflammatory and oxidative stress pathways. For example, reductions in Lactobacillus - an organism with antioxidant and anti-inflammatory properties - may contribute to lower SOD/T-AOC activity, whereas enrichment of Bacteroidetes and Streptococcus species has been linked to enhanced cytokine production. These biochemical-microbial interactions may partly explain the altered biochemical profiles observed in SLE patients with renal damage.

This study did not include several clinically relevant variables, such as disease duration, SLEDAI scores, and detailed medication exposure (e.g., glucocorticoids, immunosuppressants). These factors may influence inflammatory and oxidative markers. Future studies should incorporate multivariate biochemical analyses adjusting for these variables.

## Conclusions

In summary, the present study demonstrates that renal damage in patients with systemic lupus erythematosus is closely associated with distinct biochemical abnormalities involving heightened inflammatory cytokine activity, impaired antioxidant defenses, and elevated urinary biomarkers indicative of renal tubular injury. These biochemical signatures- characterized by increased levels of TGF-β, IL-4, IL-17, IL-1β, and ESR, together with reduced SOD and T-AOC and markedly elevated urinary microprotein, microglobulin, and NAG - reflect key molecular pathways contributing to renal dysfunction in SLE. Additionally, alterations in oral and intestinal microflora may further influence these biochemical processes, suggesting a potential link between microbial dysbiosis and biochemical mechanisms of renal injury. The combined biochemical and microbial findings provide valuable insight into the pathophysiology of SLE-associated renal involvement. More importantly, the biochemical markers evaluated in this study hold significant promise for early detection, monitoring, and clinical assessment of renal damage in SLE patients. Future studies incorporating microbial metabolites and mechanistic biochemical pathways will help further clarify the complex interactions underlying disease progression.

## Dodatak

### Funding

This work was supported by the Project of Natural Science Foundation of Heilongjiang Province (LH2022H073).

### Conflict of interest statement

All the authors declare that they have no conflict of interest in this work.
